# Brain function abnormalities and neuroinflammation in people living with HIV-associated anxiety disorders

**DOI:** 10.3389/fpsyt.2024.1336233

**Published:** 2024-03-18

**Authors:** Yunzhu Shan, Guangqiang Sun, Jiahao Ji, Zhen Li, Xue Chen, Xin Zhang, Yundong Ma, Yang Zhang, Tong Zhang, Yulin Zhang

**Affiliations:** ^1^ Center for Infectious Diseases, Beijing Youan Hospital, Capital Medical University, Beijing, China; ^2^ Beijing Key Laboratory of Mental Disorders, National Clinical Research Center for Mental Disorders & National Center for Mental Disorders, Beijing Anding Hospital, Capital Medical University, Beijing, China; ^3^ Advanced Innovation Center for Human Brain Protection, Capital Medical University, Beijing, China; ^4^ Beijing Key Laboratory of HIV/AIDS Research, Beijing, China; ^5^ Beijing Institute of Sexually Transmitted Disease Prevention and Control, Beijing, China; ^6^ Department of Respiratory and Critical Care Medicine, Beijing Youan Hospital, Capital Medical University, Beijing, China

**Keywords:** anxiety disorders, multimodal magnetic resonance, neuroimmune, human immunodeficiency virus (HIV), inflammation

## Abstract

**Background:**

People living with HIV (PLWH) exhibits an increased susceptibility to anxiety disorders, concomitant with heightened vulnerability to aberrant immune activation and inflammatory responses, and endocrine dysfunction. There exists a dearth of scholarly investigations pertaining to the neurological, immune, and endocrine dimensions of HIV-associated anxiety disorders.

**Method:**

This study aimed to compare a group of 16 individuals diagnosed with HIV-associated anxiety disorders (HIV ANXs) according to the Diagnostic and statistical manual of mental disorders (5th ed.), with a HIV individual control group (HIV control) of 49 PLWH without mental disorders. Muti-modal magnetic resonance was employed to assess the brain function and structure of both groups. Seed-based functional connectivity (FC) was used to assess the regional intrinsic brain activity and the influence of regional disturbances on FC with other brain regions. Peripheral blood cytokines and chemokines concentrations were measured using liquid chip and ELISA.

**Results:**

Amplitude of low-frequency fluctuations in the right inferior temporal gyrus (ITG) was increased. There is a significant decreased regional homogeneity in HIV ANXs in the right superior occipital gyrus (SOG). The right ITG and the right SOG were separately set as the seed brain region of interest (ROI 1 and ROI 2) to be analyzed the FC. FC decreased in HIV ANXs between ROI1 and the right middle occipital gyrus, the right SOG, FC between ROI2 and left ITG increased in HIV ANXs. No significant structural difference was found between two groups. Pro-inflammatory chemokines showed higher levels in the HIV ANXs. Pro-inflammatory cytokines, neurotrophic factors, and endocrine factors were significantly correlated with alterations in brain function.

**Conclusion:**

This study suggests that patients with HIV-associated anxiety disorders may exhibit abnormalities in neurologic, immune, and endocrine functioning. Consequently, it is imperative to implement additional screening and intervention measures for anxiety disorders among PLWH.

## Introduction

Anxiety disorders exhibit a high prevalence in the context of human immunodeficiency virus (HIV)-associated neuropsychiatric impairments, and manifest with greater frequency among people living with HIV (PLWH) as compared to HIV-negative individuals (1). The expansion of antiretroviral therapy (ART) has significantly increased the life expectancy of PLWH (2). However, numerous adverse effects can arise from ART, encompassing neuropsychiatric, metabolic, gastrointestinal, and cardiac complications. Due to potential factors such as social prejudice, compromised immune function, PLWH is also vulnerable to neuropsychiatric disorders. Among the various HIV-associated neuropsychiatric disorders, anxiety disorders are prevalent. Anxiety disorders are characterized as conditions marked by excessive anxiety and fear, accompanied by associated behavioral disturbances (3). In 2019, a staggering 301 million individuals were afflicted by anxiety disorders (4). A review revealed the median rate of anxiety disorders diagnoses in PLWH were 22.85% based on diagnostic interview (1). The prolonged survival of PLWH may be adversely affected by HIV associated neurocognitive disorders (HAND) and psychiatric disorders, in addition to challenges related to ART regimens and patient adherence, as well as treatment failures. Consequently, the overall quality of life and prognosis for PLWH have been significantly compromised.

PLWH and those diagnosed with anxiety disorders have been reported to exhibit abnormalities in brain structure and function. In PLWH who have achieved viral suppression, chronic inflammation in the central nervous system (CNS) and the presence of viral reservoirs in the CNS may potentially contribute to neuropsychological impairment during the chronic stage of infection. Previous studies have documented reductions in gray matter volume in the frontal and parietal cortices, the transitional cortex of the insula and cingulum, as well as subcortical structures including the basal ganglia, thalamus, and hippocampus in virally suppressed individuals living with HIV compared to uninfected controls (5–8). Prior studies have primarily examined HAND within the realm of HIV-associated neuropsychiatric disorders (9), while the investigation for neuroimaging of HIV-associated anxiety disorders remains limited. Numerous studies have extensively explored the utilization of brain imaging techniques to investigate anxiety disorders in normal population. Previous meta-analysis (10) showed that significant decrease in spontaneous brain activity in the right putamen, the right orbital inferior frontal gyrus, and the right temporal pole was observed in patients with anxiety disorders. Xu et al. (11) revealed that hypo-connectivity was found within and between regions, which included consistent dysregulations of affective and cognitive control related networks over networks related to emotion processing in anxiety and anxiety disorders. Structural alterations were observed in the amygdala, nucleus accumbens, bed nucleus of the stria terminalis, and regions of the prefrontal cortex, as well as the insula and parietal cortex (12). Controversially, some studies (13, 14) neglected to evaluate the existence of a formal diagnosis or relied solely on anxiety scale ratings to include participants. To enhance the precision of the findings, the diagnosis of anxiety disorders in this investigation was predicated on the structured clinical interview for the Diagnostic and statistical manual of mental disorders (DSM) (SCID) (3).

Both HIV infection and anxiety disorders have been found to induce neurological, immune, and endocrine dysfunction within the human body (15–18). The current understanding of inflammation in the field of psychiatry indicates that the perturbation of the immune system triggered by infection may contribute to the development of psychopathology. This not only adds to the psychological stress experienced by individuals enduring a potentially life-threatening illness, but also contributes to inflammation associated with stress (19). Furthermore, the interaction between the innate and adaptive immune systems and neurotransmitters has been identified as a mechanism that underlies mood disorders, psychosis, and anxiety disorders (15). The disruption of biological stress pathways, such as the Hypothalamic Pituitary-Adrenal (HPA) axis and Sympathetic-Adrenal-Medullary axis, by HIV infection is widely acknowledged. It is also recognized that chronic anxiety, which utilizes these same pathways, may play a role in the persistence of chronic inflammation and heightened anxiety symptoms over extended durations (20, 21). There are few studies on neuroinflammation in HIV-associated anxiety disorders, so in this study, we attempt to examine whether PLWH with anxiety disorders exhibit a distinct neuroinflammatory profile.

Despite extensive investigation of magnetic resonance imaging (MRI) in various illnesses, there is a lack of research regarding the particular alterations in brain structure and function that arise after the onset of anxiety disorders PLWH. The objective of this study was to address the current void in neuroimaging research on HIV-associated anxiety disorders by conducting a comparative analysis of imaging disparities between patients with HIV-associated anxiety disorders and HIV-infected individuals without psychiatric disorders. Additionally, we sought to examine the association between these alterations in brain imaging and clinical indicators. The findings of this study may provide a theoretical foundation for the early detection of anxiety disorders associated with HIV, based on the afore mentioned results.

## Materials and methods

### Participants

This study has been approved by the Ethics Committee of Beijing Youan Hospital, Capital Medical University (2023/057). Each participant has been informed about the purpose of the study, and a written informed consent was obtained. For experiments involving humans, the research was conducted in accordance with the Declaration of Helsinki of the World Medical Association revised in 2013.

Between May 2022 and November 2022, a total of 109 PLWH with proof of HIV infection were recruited. Patients presenting with opportunistic infection, autoimmune diseases, epilepsy, traumatic brain injury, substance use, undergoing suppressive therapy, or other severe disease were excluded from the study. All the patients treated with ART with undetectable serum viral loads. All participants consistently adhered to their ART drugs, with no recent documented modifications to their ART regimens. The clinical characteristics of the subjects (including demographics, CD4^+^ T cell count, HIV load, date of HIV infection, duration of HIV infection, date of ART initiation, duration of ART treatment, and type of ART drugs) were obtained from the medical records of the AIDS research cohort at Beijing Youan Hospital. Brain imaging data, peripheral blood specimens, and SCID were collected from each participant. Based on the SCID results, the subjects were categorized into two groups: HIV-infected individuals without any mental disorders, serving as the HIV control group (HIV control), and HIV-infected individuals diagnosed with anxiety disorders, forming the HIV-associated anxiety disorders group (HIV ANXs). Participants who did not have HIV-associated anxiety disorders but had other mental disorders were excluded from the study. Peripheral blood samples were collected from each participant in the morning (8-10 am) after fasting for at least 8 hours. For each participant, plasma and peripheral blood mononuclear cell (PBMC) samples were stored at −80°C and in liquid nitrogen tanks, respectively.

### Measurement of anxiety disorders

Anxiety was initially evaluated by using the self-rating depression scale (SDS) and self-rating anxiety scale (SAS). Then an experienced psychiatrist conducted this fully structured interview for lay interviewers to measure anxiety, mood, substance use, and personality disorders according to the DSM-V.

### Neuroimaging

#### Image acquisition

Resting-state functional MRI data were acquired using a 1.5 T Philips MRI Scanner at the Beijing Second Hospital. Participants were instructed to lie supine, maintain immobility, close their eyes, and remain awake. To minimize head movement, soft earplugs were employed. The resting-state functional images were obtained using the echo planar imaging (EPI) sequence, with the following parameters: repetition time/echo time (TR/TE) 4000/25 ms, 40 slices, 64*64 matrix, 90°flip angle, 2.8 mm slice thickness, no gap, and 102 volumes. For 3D-T1WI: TR/TE = 2500/2.98 ms, flip angle = 7°, matrix = 64 × 64, field of view (FOV) = 256 mm × 256 mm, slice thickness = 1 mm, slice number = 48, slices =192, scanning time 6 min 3 s.

#### Data preprocessing

Data preprocessing was conducted in Matlab R2013b by using SPM12. The first 10 images were discarded. Functional images were motion corrected using the realign function. Among the participants, 9 were excluded due to the maximum displacements exceeding 2 mm in the x, y, or z axis, or angular rotations exceeding 2 degrees, after correction for head motion. The standard DARTEL template in MNI space was used to drive DARTEL normalization. Then regression of nuisance covariates was performed. After that, the functional MRI (fMRI) data were then subjected to temporal band-pass filtering (0.01–0.08 Hz) and linear detrending. Spurious covariates, such as the 6-head motion parameters obtained through rigid body correction, were eliminated.

#### Amplitude of low-frequency fluctuations and fractional ALFF analysis

ALFF was calculated by the Resting-State fMRI Data Analysis Toolkit (RESTplus) version 1.8. The ALFF value was calculated for each voxel, which was further divided by the global mean value for standardization. The standardized ALFF value is divided by the ALFF value over the entire frequency band to obtain the standardized fALFF value for each voxel.

#### Regional homogeneity analysis

ReHo analysis was also conducted by RESTplus version 1.2. The Kendall coefficient of consistency (KCC) was calculated between the time series of a given voxel and the time series of its last 26 voxels. The ReHo maps were standardized by dividing the KCC of each voxel by the mean KCC of the whole brain. The resulting imaging data were then spatially smoothed with a Gaussian kernel of 4 mm full-width at half-maximum.

#### Gray matter volume data processing

The GMV was calculated by SPM12. The steps include: (1) Individual structural images were segmented into three types of brain structures as gray matter, white matter, and cerebrospinal fluid; (2) Anatomical images were standardized to the Montreal Neurological Institute Brain Template by using the DARTEL toolkit; (3) Non-linear modulation was adopted to modulate the voxel values from density to volume; (4) Gaussian kernel Smoothing was conducted; (5) Brain GMV was quantitatively measured.

#### Functional connectivity analysis

The brain regions exhibiting significant differences in ALFF/fALFF, ReHo, and GMV were selected as seeds for FC analysis. Following band-pass filtering (0.01 - 0.08 Hz) and linear trend elimination, a reference time series for each seed was obtained by averaging the resting-state fMRI (rs-fMRI) time series of voxels within each seed. Pearson’s correlation coefficients were then computed voxel-wise between the time series within each region of interest (ROI) and the filtered time series in the remaining brain regions. To enhance normality, Fisher’s r-to-z transformation was applied to convert the correlation coefficients in each voxel into z-scores.

### Cytokine, chemokine assay

#### Luminex® xMAP® technology

We conducted a comprehensive cytokine/chemokine assay in the plasma samples of various groups using the EMD Millipore’s MILLIPLEX® MAP Human Cytokine/Chemokine/Growth Factor Panel A MAGNETIC BEAD PANEL 96-Well Plate Assay, which is based on the cutting-edge Luminex® xMAP® technology.

We employed capture antibody-coupled magnetic beads to selectively bind the target analytes in the plasma. The plate was wrapped with foil and incubated it with gentle agitation on a plate shaker overnight, maintaining a controlled temperature of 2–8 °C. After the incubation period, the plate was washed three times. Subsequently, we incubated the plate with detection reagents, again employing gentle agitation on a plate shaker, for a duration of 1 hour at room temperature (24 °C). Finally, we utilized the advanced Luminex® 200™, HTS, FLEXMAP 3D® software to run the plate and analyzed the resulting median fluorescent intensity (MFI) data. This analysis was conducted by a sophisticated 5-parameter logistic or spline curve fitting method, ensuring accurate interpretation of the obtained results.

#### Enzyme-linked immunosorbent assay

Endocrine hormones and neuro-factors concentration was determined by the following ELISA kits: Nerve growth factor (NGF), Human beta-NGF ELISA 96T; glial cell-derived neurotrophic factor (GDNF), Human GDNF ELISA 96T; insulin-like growth factor 1 (IGF-1), Human IGF-1 ELISA 96T; cortisol: Human Cortisol EIA 96T; corticotropin-releasing hormone (CRH), Human CRHBP ELISA 96T; adrenocorticotropic hormone (ACTH), Human Adrenocorticotropic Hormone EIA 96T. All assays were performed according to the manufacturers’ protocols.

### Statistical analyses

The differences in ALFF/fALFF, ReHo, FC, GMV between HIV control and HIV ANXs were performed with a two-sample t-test using SPM12. The age, CD4 count at initiation of ART, recent CD4 counts were treated as covariates. The topological false discover rate (FDR) was used for multiple comparisons correction with the significance threshold 0.05. Differences in demographic information between HIV control and HIV ANXs were examined with IBM SPSS 27.0 (IBM Inc. Armonk, NY, USA). The Z score outside the range from -3 to 3 were considered outliers. The VIF value was used to evaluate multicollinearity among the variables, with a value of > 10 representing high multicollinearity. Assumptions of normality and homogeneity of variance were assessed using Shapiro-Wilk’s test and Levine’s test. Mann-Whitney U test, multivariate analysis of variance (MANOVA), χ2 test, or Fisher’s exact test were used to compare the differences in clinical characteristics, cytokines, and neuroendocrine factors between two groups. The significance threshold was set to 0.05, two-tailed. Pearson or Spearman correlation analyses were performed to evaluate the relationship between brain functional, structural changes (ALFF/fALFF, ReHo, FC, and GMV) and clinical characteristic and peripheral blood indicators.

## Results

### Demographic and clinical characteristics

All subjects included in this study were male (n = 65), a total of 49 people were categorized into HIV control and 16 into HIV ANXs based on the SCID. There was a significant difference of age between HIV controls and HIV ANXs (P = 0.050), the mean age was 35.53 ± 7.86 years in the HIV controls and 40.13 ± 7.74 years in HIV ANXs. A significant increase was observed in HIV ANXs in the initial CD4^+^ T cell count at the initiation of antiviral therapy (P = 0.041), and recent CD4^+^ T cell count (P = 0.013), compared with HIV control (**Table 1**).

**Table 1:** Demographic and clinical characteristics of all subject.

Demographic and clinical data	HIV control (n = 49)	HIV ANXs (n = 16)	*P-*value
Age, years, mean (SD)	35.53 (7.86)	40.13 (7.74)	0.050*^a^
Duration of education, years, median (IQR)	16.00 (13.50, 16.00)	15.50 (11.25, 16.00)	0.449^b^
BMI, kg/m^2^, mean (SD)	22.54 (2.88)	23.33 (3.99)	0.475^a^
CD4^+^ T cell count			
Initial CD4 count, count/ mm^3^, mean (SD)	342.47 (213.55)	466.19 (211.68)	0.062^a^
CD4 count at initiation of ART, count/mm3, mean (SD)	339.91 (213.08)	474.56 (218.13)	0.041* ^a^
Recent CD4 count, count/ mm3, median (IQR)	564.00 (407.00, 779.00)	773.00 (550.00, 995.75)	0.013*^b^
CD4/CD8			
Initial CD4/CD8, median (IQR)	0.36 (0.16, 0.44)	0.38 (0.27, 0.53)	0.359^b^
CD4/CD8 at initiation of ART, median (IQR)	0.37 (0.22, 0.46)	0.36 (0.23, 0.51)	0.637^b^
Recent CD4/CD8, mean (SD)	0.711 (0.35)	0.733 (0.26)	0.801^a^
HIV load			
Initial HIV load (copies/mL), median (IQR)	9777.00 (3629.50, 48586.25)	10710.00 (4500.00, 36945.00)	0.929^a^
HIV load at initiation of ART, copies/mL, median (IQR)	10918.00 (3648.50, 66687.00)	10537.50 (4843.00, 34601.25)	0.929^b^
Time to initiate ART after HIV discovery, months, median (IQR)	0.50 (0.40, 1.70)	0.55 (0.43, 1.00)	0.565^b^
Duration of ART, months, mean (SD)	64.13 (38.40)	75.60 (49.22)	0.377^a^
Duration of HIV infection, months, mean (SD)	70.66 (42.38)	81.71 (45.35)	0.398^a^
Initial ART drugs type			0.193^c^
INSTIs, n (%)	10 (20.41)	1 (6.25)	
Other types of ART drugs, n (%)	39 (79.59)	15 (93.75)	
Recent ART drugs type			0.339^c^
INSTIs, n (%)	34 (69.39)	9 (56.25)	
Other types of ART drugs, n (%)	15 (30.61)	7 (43.75)	
SAS, median (IQR)	30.00 (24.00, 34.00)	41.50 (36.00, 46.75)	<0.0001 ****^b^
SDS, median (IQR)	30 (26.00, 35.50)	44.00 (35.00, 48.75)	<0.001 ***^b^

HIV control: HIV individual control group; HIV ANXs: HIV-associated anxiety disorders group; BMI: body mass index; INSTIs: Integrase strand transfer inhibitors; SAS: Self-rating Anxiety Scale; SDS: Self-rating Depression Scale; *: P < 0.05; ***: P < 0.001; ****: P < 0.0001; ^a^T-test; ^b^Mann-Whitney U test; ^c^χ2 test; IQR, Interquartile range; SD, Standard deviation. The Shapiro-Wilk test was performed to assess the normal distribution of variables.

### Neuroimaging differences

#### ALFF/fALFF and ReHo

**Figure 1:**
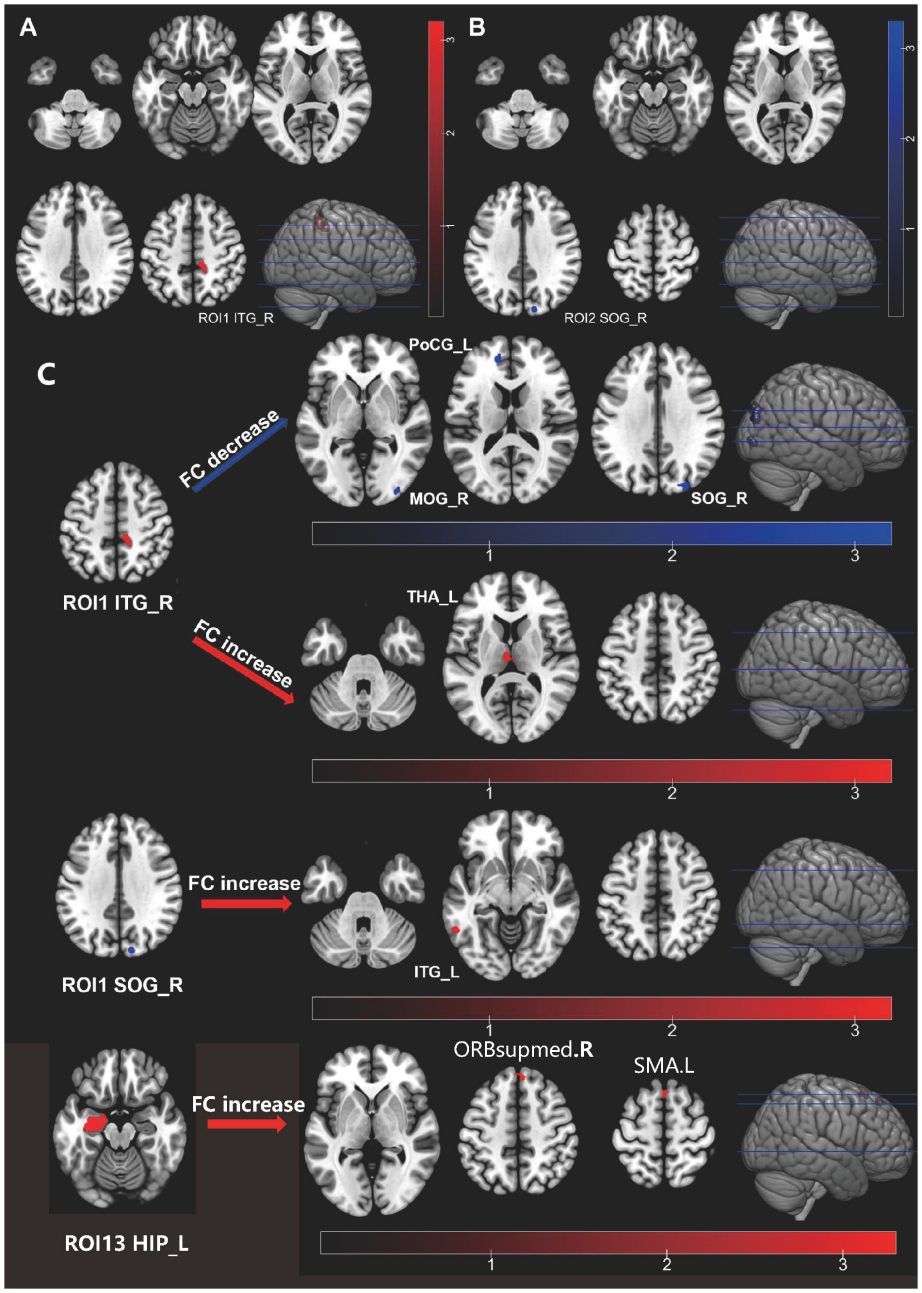
Individuals with HIV-associated anxiety disorders exhibit abnormal functional activity and functional connectivity in their brain. **(A)** ALFF alteration in HIV ANXS; **(B)** ReHo alteration in HIV ANXs; **(C)** FC alteration in HIV ANXs. Red regions show hyperactivity, and blue regions show hypoactivity in HIV ANXs; The difference was calculated by two-tailed two-sample t-test. ALFF, amplitude of low-frequency fluctuations; HIV ANXs, patients with HIV-associated anxiety disorders. HIV control, PLWH without mental disorders controls; Temporal_Inf_R, the right inferior temporal gyrus; Occipital_Sup_R, the right superior occipital gyrus; Occipital_Mid_R, the right middle occipital gyrus; Occipital_Sup_R, the right superior occipital gyrus; Postcentral_L, the left postcentral gyrus; Thalamus_L, the left thalamus; Temporal_Inf_L, the left inferior temporal gyrus; ORBsupmed.R, the right superior frontal gyrus, medial orbital; SMA.L, the left Supplementary Motor Area.

We observed alterations in ALFF and ReHo between the HIV ANXs and HIV control. A two-sample t-test was conducted, revealing an increase in ALFF in the right inferior temporal gyrus (ITG) (**Figure 1A**; **Supplementary Table 1**). Considering the existence of systematic sample difference, age, CD4 count at initiation of ART and recent CD4 counts were treated as covariates. The significant difference in ALFF remained even after correcting for these covariates. However, no significant fALFF was found between two groups. The results also showed a significant decrease in ReHo in the right superior occipital gyrus (SOG) of the HIV ANXs (**Figure 1B**; **Supplementary Table 1**). Unfortunately, ReHo difference does not exist after correction for covariates.

#### GMV

GMV did not differ significantly between HIV control and HIV ANXs.

#### FC

We set the brain region with abnormalities in ALFF and ReHo as the seed brain region of interest (ROI) (the right ITG as ROI1, the right SOG as ROI2), and analyzed FC of this brain region to the whole-brain voxels (**Figure 1C**; **Supplementary Table 2**). We found that FC decreased in HIV ANXs between ROI1 and the right middle occipital gyrus (MOG), the right SOG, the left postcentral gyrus (PoCG), and FC increased between ROI1 and left thalamus (THA), when compared with HIV control. In addition, FC between ROI2 and left ITG increased in HIV ANXs.

Given the potential for inflated findings when only significant regions are included in a follow-up analysis (22), and considering the early predilection for subcortical regions in HIV, we then set frontal and their corresponding striatal regions, THA regions, and hippocampus (HIP) regions as ROI (ROI 3 – 22, as shown in **Supplementary Table 3**). The left HIP was set as ROI13. We found that FC increased in HIV ANXs between ROI13 and right superior frontal gyrus, medial (SFGmed), left Supplementary Motor Area (SMA). No other differences in FC were observed between the two groups.

### Peripheral blood indicators difference

There was difference of inflammatory indicators between the two groups. Outliers with a |Z score| greater than 3 were excluded in interleukin (IL)-13 and tumor necrosis factor (TNF)-β. We found that eotaxin, interleukin IL-4, in HIV ANXs were significantly higher than HIV control. (**Table 2**). MANOVA was performed with cytokines, neurotrophic factors, and endocrine factors as independent variables, and anxiety disorders diagnosis based on the DSM-5 as the dependent variable. After removing variables that did not meet the criteria for homogeneity of variance and exhibited multicollinearity, 14 indicators were included in MANOVA. However, the results did not reveal any between-group differences (**Supplementary Table 4**).

**Table 2:** Cytokines and chemokines differences between HIV control and HIV ANXs.

Inflammatory, endocrine and neurotrophic indicators, pg/mL, median (IQR)	HIV control (n = 49)	HIV ANXs (n = 16)	*P*-value
Cytokines			
Pro-inflammatory			
IL-1beta	10.29 (7.60, 12.43)	10.23 (8.52, 16.91)	0.393
IL-7	4.91 (3.27, 6.64)	5.43 (4.36, 6.72)	0.286
IL-8	1.89 (1.12, 2.70)	2.25 (1.40, 3.21)	0.193
IL-12 (p40)	50.32 (33.50, 60.00)	50.32 (46.38, 78.30)	0.180
IL-15	2.82 (2.30, 3.34)	2.87 (2.10, 4.53)	0.664
IL-17A	3.89 (1.35, 5.93)	5.93 (0.94, 11.75)	0.266
IL-17E/IL-25	589.73 (507.30, 677.26)	604.74 (465.42, 912.58)	0.492
IL-17F	17.01 (13.31, 20.86)	18.17 (12.75, 30.00)	0.273
IL-18	170.11 (125.80, 299.57)	122.72 (102.87, 310.55)	0.290
IP-10	219.45 (179.46, 271.77)	244.25 (201.60, 275.18)	0.235
IFN-alpha2	29.74 (79.80, 38.02)	36.59 (21.73, 57.72)	0.148
IFN-gamma	1.01 (0.87, 1.54)	1.16 (0.78, 2.90)	0.259
TNF-beta	0.21 (0.06, 0.86)	0.49 (0.13, 5.78)^a^	0.065
G-CSF	57.87 (39.69, 72.57)	70.79 (50.87, 120.44)	0.150
M-CSF	37.06 (27.38, 53.53)	37.66 (23.88, 72.36)	0.527
sCD40L	2889.00 (2006.50, 2889.00)	3467.50 (2239.75, 4673.25)	0.300
VEGF-A	59.35 (36.82, 108.52)	100.93 (48.26, 147.16)	0.069
Anti-inflammatory			
IL-1alpha	4.61 (3.18, 8.24)	5.75 (3.52, 16.65)	0.360
IL-1RA	10.03 (7.60, 13.75)	14.31 (6.16, 20.88)	0.469
IL-3	0.75 (0.44, 1.13)	0.72 (0.43, 2.16)	0.583
IL-4	2.25 (1.76, 3.01)	3.27 (1.87, 4.96)	0.035*
IL-5	4.55 (3.59, 5.64)	5.50 (3.41, 8.66)	0.389
IL-10	4.16 (2.65, 6.72)	4.76 (2.65, 6.82)	0.778
IL-13	21.84 (9.68, 33.23)	28.85 (20.61, 63.10) ^a^	0.074
TGF-alpha	3.26 (2.19, 4.37)	3.68 (1.80, 7.51)	0.289
IL-6	0.72 (0.50, 1.04)	0.68 (0.42, 1.91)	0.720
TNF-alpha	18.33 (12.23, 23.81)	19.16 (15.73, 37.69)	0.217
IL-9	20.57 (15.41, 25.65)	23.34 (18.26, 34.06)	0.163
Chemokines			
Eotaxin	46.01 (41.61, 52.84)	50.88 (47.18, 59.75)	0.048*
MCP-1	153.83 (135.66, 185.78)	139.11 (114.49, 195.53)	0.180
MCP-3	19.22 (13.00, 14.66)	21.00 (14.47, 37.80)	0.209
MIP-1alpha	20.57 (14.82, 27.82)	23.52 (14.13, 48.08)	0.244
MIP-1 beta	69.12 (55.47, 86.68)	75.72 (67.42)	0.133
MIG	1519.00 (1258.00, 1942.00)	1881.00 (1463.00, 2596.00)	0.057
Endocrine factors			
ACTH	0.62 (0.31, 1.57)	0.8 (0.37, 2.80)	0.404
CORT	9.65 (7.31, 15.80)	9.01 (6.24, 12.60)	0.468
CRH	2.48 (2.10, 3.31)	2.59 (2.26, 3.60)	0.939
Neurotrophic factors			
FGF-2	156.52 (120.11, 186.71)	173.31 (124.04, 274.04)	0.286
PDGF-AA	2580.00 (1861.00, 3360.00)	3022.00 (2517.00, 3983.25)	0.100
PDGF-AB/BB	27129.00 (20848.00, 34191.50)	31393.00 (22772.00, 40814.50)	0.337
BNGF	98.90 (56.79, 246.88)	1756.45 (190.37, 3444.35)	0.079
IGF-1	0.26 (0.14, 0.67)		0.157
BDNF	0.59 (0.48, 0.68)	0.59 (0.42, 0.81)	0.915

HIV control, HIV individual control group; HIV ANXs, HIV-associated anxiety disorders group; IL, interleukin; IFN, interferon; TNF, tumor necrosis factor; G/M-CSF, granulocyte/macrophage colony-stimulating factor; VEGF, vascular endothelial growth factor; TGF, transforming growth factor; MCP, monocyte chemotactic protein; MIP, macrophage inflammatory protein; ACTH, adrenocorticotropic-hormone; CORT, cortisol; CRH, corticotropin releasing hormone; FGF, fibroblast growth factor; PDGF, platelet-derived growth factor; BNGF, human nerve growth factor; IGF, insulin-like growth factor; BDNF, brain-derived neurotrophic factor; *, p<0.05; ^a^after removing one outlier, n = 15.

### Associations between brain function alterations and clinical characteristics

**Figure 2:**
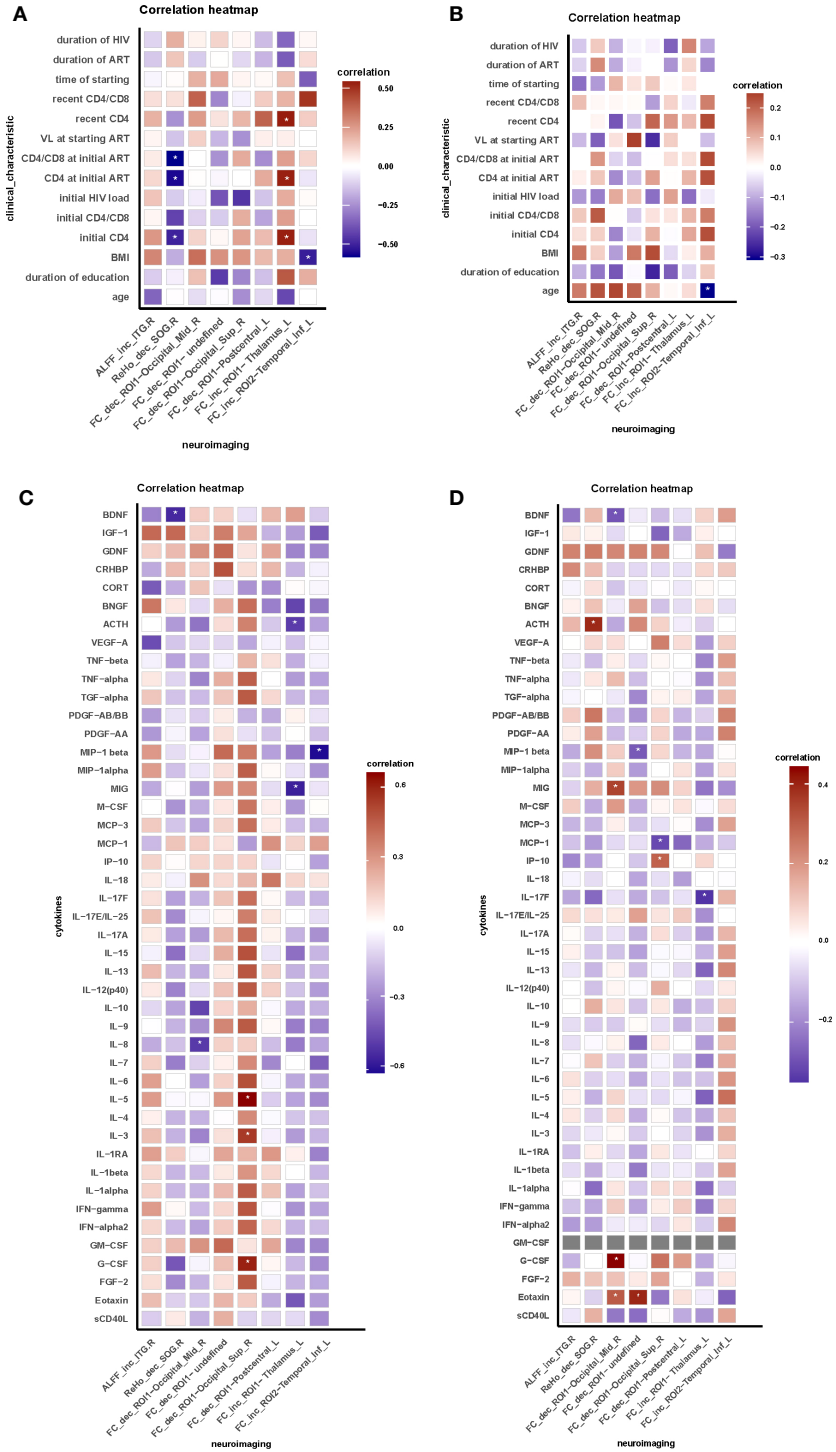
Brain functional abnormalities are correlated with clinical characteristic and peripheral blood indicators in HIV-associated anxiety disorders. **(A)** Correlation between neuroimaging and clinical characteristic in HIV ANXs; **(B)** Correlation between neuroimaging and clinical characteristic in HIV control; **(C)** Correlation between neuroimaging and peripheral blood indicators in HIV ANXs; **(D)** Correlation between neuroimaging and peripheral blood indicators in HIV control. Correlation analyzed by Spearman’s correlation. Data are shown as media and Interquartile range. *p<0.05. FC, functional connectivity; ROI, region of interest; HIV ANXs, patients with HIV-associated anxiety disorders. HIV control, PLWH without mental disorders controls; Temporal_Inf_R, the right inferior temporal gyrus; Occipital_Sup_R, the right superior occipital gyrus. Occipital_Mid_R, the right middle occipital gyrus; Occipital_Sup_R, the right superior occipital gyrus; Postcentral_L, the left postcentral gyrus; Thalamus_L, the left thalamus; Temporal_Inf_L, the left inferior temporal gyrus.

In HIV ANXs, we conducted a correlation analysis (**Figures 2A, C**). The results indicated that, in HIV ANXs, decrease of ReHo in right SOG is negatively correlated with initial CD4^+^ T cell count, CD4^+^ T cell count at initiation of ART, BDNF. Decrease of FC between ROI1 and right MOG is negatively correlated with IL-8. G-CSF granulocyte colony-stimulating factor (G-CSF), IL-3, IL-5 are positively correlated with a decrease in FC between ROI1 and the right SOG. Increase of FC between ROI1 and the left THA is negatively correlated with MIG, CD4^+^ T cell count, and positively correlated with ACTH. Furthermore, MIP-1beta is correlated with the increase of FC between ROI2 and the left ITG. Correlation analysis in HIV control was shown in **Figures 2B, D**.

## Discussion

This article offers a thorough analysis of the neuroimmune profile in HIV-associated anxiety disorders through the evaluation of multimodal MRI, neuro-immunity, and endocrine functioning manifestations. First, significant disparities were identified, in terms of immune status such as CD4^+^ T cell count at the initiation of ART, and recent CD4^+^ T cell count, all of which were notably higher in HIV ANXs. Furthermore, it was observed that HIV ANXs exhibit alterations in brain activity and functional connectivity, while the structural difference was not observed. Additionally, HIV-associated anxiety disorders are linked to a heightened systemic inflammatory response in comparison to HIV control. Moreover, our research has revealed noteworthy correlations between aberrant brain activity, functional connectivity, and immune, inflammatory, endocrine dysfunction in the context of HIV-associated anxiety disorders.

ALFF and ReHo were observed to be significantly different in HIV ANXs, while no differences were found in GMV between two groups. There is a consensus in previous studies regarding neuroimaging studies of anxiety disorders, with the amygdala emerging as a key structure implicated in fear and anxiety responses, consistently showing activation in anxiety-inducing contexts. In addition to the amygdala, the insula and anterior cingulate cortex are also considered critical components of the fear network (23). In our study, the analysis of ALFF/fALFF reveals that the HIV-associated anxiety disorders exhibit significantly elevated levels of ALFF in the right ITG, indicating hyperactivity. The middle and inferior temporal gyrus are recognized for their crucial involvement in both the dorsal and ventral visual pathways, as well as in multimodal and higher sensory processing (24). Functionally, the ITG plays a role in visual perception (25) and is associated with the ventral visual pathway (26). Additionally, the ITG exhibits connections with the amygdala and orbitofrontal cortex, which are primarily implicated in the processing of negative emotions (27). In our study, the calculation of ReHo revealed a significant decrease in the right SOG, which is involved in visual processing. This finding aligns with a previous fMRI study that demonstrated activation of the visual cortex during exposure to social threat in social anxiety disorder (SAD) (28). Previous studies have also suggested a decrease in ReHo in the inferior frontal gyrus (IFG) (29), orbital middle temporal gyrus, middle frontal gyrus, and anterior cingulate (30) in patients with generalized anxiety disorder (GAD). This implies that similar visual processing abnormalities might be involved in HIV-associated anxiety disorders. Moreover, GMV difference was not observed in our study. This differs from the previous findings, a meta-analysis revealed unique diminutions in GMV for anxiety disorders (31), the findings indicated that patients with anxiety disorders exhibited significantly reduced GMV, specifically in left STG extending to IFG. Regarding HIV-associated neuropsychiatric disorders, the current focus of investigations lies in HAND, where diffuse cerebral atrophy and cerebral white matter abnormalities emerge as the prevailing manifestations in early MRI. Various degrees of HIV-associated cognitive impairment are associated with varying levels of alteration in GMV (32). Additionally, the presence of white matter abnormalities serves as an indication of HIV-associated encephalitis (33). Consequently, in order to reach a conclusion of neuroimaging features in HIV-associated anxiety disorders, it is imperative to undertake additional research studies.

The alteration of FC, which enables the examination of intrinsic neural circuits and their functional states, was also observed in HIV ANXs (34). Prior research has suggested a correlation between anxiety and alterations in the structural and functional connectivity of the hypothesized fear and limbic cortico-striato-thalamocortical circuits (35). The amygdala and insula have been identified as key regions of interest for investigating the functional connectivity in individuals with anxiety disorders, given their significant roles in emotion regulation. Recent findings of functional connectivity studies suggested a correlation between anxiety disorders and changes in connectivity patterns between the amygdala and frontal brain regions, as well as within the default mode network (36, 37). Specifically, alterations in connectivity were observed predominantly in the frontal, occipital, temporal, and parietal-(pre)motor regions (37, 38), indicating disrupted connections across various brain networks in individuals with anxiety disorders. In this study, we calculated resting-state functional connectivity maps, using the right ITG right SOG, frontal and their corresponding striatal regions, THA regions, and HIP regions as seed ROIs to assess differences in the crosstalk among brain regions. Our study showed decreased FC in HIV ANXs between right ITG and the right MOG, the right SOG, the left PoCG, increased FC between the right SOG and left ITG increased in HIV ANXs, and increased FC between the right ITG and left THA. The decrease of FC may be a manifestation of brain impairment. However, it is unclear whether increased FC is a pathological or a early compensatory mechanism in HIV-associated anxiety disorders. We also found increased FC in the right SOG and the right ITG. As previously mentioned, visual cortex is activated during exposure to social threat in SAD (28), this mechanism may also be present in HIV-associated anxiety disorders. Hence, HIV-associated anxiety disorders may activate the visual cortex, or it may be a compensatory mechanism resulting in increased FC of the right ITG and right SOG. Previous research has hypothesized that in GAD patients, amygdalo-fronto-parietal coupling reflects “habituated engagement of cognitive controls to regulate excessive anxiety” (39). A recent meta-analysis (40) based on stringent diagnostic criteria for anxiety disorders revealed that, studies using the amygdala as seed regions did show altered connectivity with the medial prefrontal cortex. Additional studies will be required to support this hypothesis whether this amygdala-related feature is also present in HIV-related anxiety disorders.

The study did not find a significant difference in inflammation response between the two groups, despite previous research indicating the presence of chronic inflammation in both HIV infection and anxiety disorders. However, the present study only identified variations in eotaxin and IL-4 levels between the groups. Given the limitations of a small sample size, the absence of a healthy control group, and the detection of significant differences in only two out of several cytokines analyzed, the findings may not fully capture the inflammatory response in both the HIV controls and HIV ANXs. Based on these, we propose the following interpretation of the results examined. The HIV ANXs consistently exhibited higher levels of proinflammatory chemokine (eotaxin). Furthermore, the anti-inflammatory cytokine IL-4 were found to be elevated in HIV ANXs. These findings indicate that patients with HIV-associated anxiety disorders have heightened levels of inflammation response. The compensatory elevation of IL-4, anti-inflammatory cytokine, may serve as a regulatory mechanism. Chemokines play a crucial role in initiating the immune response by selectively recruiting specific subpopulations of immune cells to the location of antigen presentation (41). Eotaxin, a chemokine with pro-inflammatory properties, is also implicated in the regulation of inflammation. There is preliminary evidence that anxiety disorders are associated with an increase in pro-inflammatory markers. The existing findings indicate that GAD and panic disorders (PD) in particular are associated with a pro-inflammatory state, which is evidenced by elevated peripheral cytokines and CRP concentrations in these diseases (42). Additionally, there is increasing evidence that pro-inflammatory markers can modulate affective behavior directly in anxiety disorders (42). Despite having distinct diagnostic criteria, anxiety disorders are characterized by excessive fear and anxiety, and therefore may have similar neurobiological features. The presence of increased inflammatory signals including cytokines, has been linked to posttraumatic stress disorder, GAD, PD, and phobias. Nevertheless, not all reports indicate a positive association between inflammation and anxiety, suggesting that other factors should be taken into account in future assessments of inflammation’s role in the progression of these disorders (such as gender, co-morbid conditions, and other sources of inflammation) (42). Persistent inflammation and immune activation are established features of chronic HIV infection (43) and effectively discriminate HIV status and comorbid risk (44). In this study, we have observed that the co-existence of chronic inflammation in individuals with HIV infection and anxiety disorders leads to enduring chronic inflammation in patients with HIV-associated anxiety disorders, as evidenced by heightened levels of pro-inflammatory cytokines. Consequently, we posited the existence of a distinct mechanism within HIV ANXs that undergoes immune and inflammatory modulation mediated by eotaxin, resulting in the modulation of inflammatory cytokine secretion. However, considering that only two indicators, eotaxin and IL-4, were present in the results of the present study that differed, we cannot clarify whether other cytokines are involved in this mechanism. Nonetheless, further experimentation is necessary to thoroughly investigate this hypothesis.

Interestingly, a significant association was observed between the modified brain function in HIV ANXs and their individual immune status, inflammation response, and endocrine dysfunction. We found that decrease of ReHo in right SOG is negatively correlated with initial CD4^+^ T cell count, CD4^+^ T cell count at initiation of ART and BDNF. This suggests that chronic immune dysregulation due to reduced CD4^+^ T cell count leads to hypoactivation of brain function in PLWH. The role of BDNF in anxiety is controversial (45), and BDNF expression was observed to be reduced in the PLWH. Therefore, correlation between BDNF and alteration of ReHo may be a characteristic manifestation of HIV-associated anxiety disorders. Besides, decrease of FC between the right ITG and right MOG is negatively correlated with IL-8, and G-CSF, IL-3, IL-5 is positively correlated with a decrease in FC between ROI1 and the right SOG. Correlation of MIP-1beta and the increased functional connectivity of right SOG was detected. These results indicate that alterations in pro-inflammatory cytokines and chemokines also affect brain functional connectivity in patients toward different directions. The temporal gyrus and occipital gyrus are both involved in visual processing. Therefore, the correlation between these two brain-regions’ functional connectivity and patients’ immune and inflammatory states, further confirms that patients with HIV-related anxiety disorders may have visual processing abnormalities. No studies have yet found abnormal thalamic function in patients with anxiety disorders. A earlier study using brain morphometry analyses in SAD are available to date, it indicated no difference in striatal structures (caudate, putamen, and thalamus) (46). Our finding suggests that increase of FC between ROI1 and the left thalamus is negatively correlated with MIG, initial CD4^+^ T cell count, though no significant difference of thalamus was observed between two groups. Furthermore, it is noteworthy that a positive correlation exists between ACTH levels and increased FC between the right ITG and the left THA. It is widely recognized that dysregulation of the HPA axis is the primary endocrine effect in HIV infection (47). Simultaneously, anxiety triggers activation of the HPA axis (17). The alterations in ACTH levels observed in HIV ANXs may be attributed to the co-morbidity of HIV infection and anxiety disorders.

To our knowledge, this is the first time to systematically collect and analyze demographic, clinical, multimodal MRI imaging, and immunological data on patients with HIV-associated anxiety disorders. We used the SCID for the diagnosis of anxiety disorders to ensure the accuracy of the diagnosis and groupings. Based on these results, we can hypothesize that patients with HIV-associated anxiety disorders develop visual processing deficits in the cerebral cortex compared to HIV-infected patients without psychiatric disorders, and that this alteration is closely related to immune status, the degree of chronic inflammation response, and endocrine dysfunction in patients. This provides a theoretical basis for early screening and advance intervention for HIV-associated anxiety disorders.

Limitations of the study and future directions for research in the field deserve attention. First, to gain a deeper understanding of HIV-associated anxiety disorders neurophysiopathology, further multimodal studies are needed. Second, despite recruiting a consistent number of comorbidity-free HIV-infected patients, we should extend our findings to larger anxiety disorders sample sizes in the future, especially for GMV analysis. However, the examination of GMV was solely confined to the alteration in gray matter volume, while numerous studies incorporate a plethora of additional analytical techniques and tools. For instance, MRI diffusion tensor imaging (DTI) enables the assessment of white matter microstructure by providing measurements of fiber organization and unhindered water motility. In forthcoming research endeavors, it is imperative to obtain a greater array of multidimensional MR data in conjunction with more widely adopted analytical methods such as FSL’s FAST and ANTs. This will facilitate a comprehensive characterization of the brain function and structure of individuals with HIV ANXs. Additional limitations of our study encompassed a limited sample size, the lack of a control group, and notable between-group variations for only two of the numerous cytokines examined, along with a total of 26 significant correlations identified in a multifactor correlation analysis. To enhance the validity of our findings, it is imperative to increase the sample size and incorporate a healthy control group in future studies. The inadequacy of the sample size in the present study underscores the need for improvement in subsequent research endeavors. While CD4 counts may not provide a comprehensive indication of immune status in HIV-infected individuals, our study did find higher CD4 counts in the HIV ANXs group, albeit not statistically significant. This finding underscores the importance of considering CD4 counts as a potential influencing factor in future research, necessitating the inclusion of more subjects matched on baseline metrics in subsequent studies. Furthermore, MRI scans were acquired at 1.5T, which may result in us being unable to acquire more detailed neuroimaging. To enhance the examination of the functional and structural attributes of the brain in HIV ANXs, it is imperative to employ more refined scanning techniques in forthcoming studies.

## Conclusion

A series of neurological, endocrine, and immune dysfunctions that occur after HIV infection may contribute to HIV-associated anxiety disorders. The present study revealed aberrations in brain function and functional connectivity among individuals with HIV-associated anxiety disorders, in comparison to PLWH without psychiatric disorders. Additionally, patients with HIV-associated anxiety disorders exhibited heightened abnormal immune dysfunction and chronic inflammatory response levels. Furthermore, a noteworthy association was identified between alterations in brain function and changes in levels of body immunity, inflammation, and endocrine within the patient cohort. Our findings have the potential to serve as a significant foundation for future research endeavors that integrate morphometric, functional, anatomical, and immunological data. This holistic approach will contribute to a comprehensive comprehension of the neural circuitry that underlies HIV-associated anxiety disorders.

## Data availability statement

The original contributions presented in the study are included in the article/**Supplementary Material**. Further inquiries can be directed to the corresponding authors.

## Ethics statement

The studies involving humans were approved by the Ethics Committee of Beijing Youan Hospital, Capital Medical University (2023/057). The studies were conducted in accordance with the local legislation and institutional requirements. The participants provided their written informed consent to participate in this study.

## Author contributions

YS: Conceptualization, Data curation, Formal analysis, Investigation, Methodology, Validation, Visualization, Writing – original draft, Writing – review & editing. GS: Conceptualization, Data curation, Formal analysis, Investigation, Methodology, Visualization, Writing – original draft, Writing – review & editing. JJ: Conceptualization, Data curation, Formal analysis, Investigation, Methodology, Visualization, Writing – original draft, Writing – review & editing, Software. ZL: Formal analysis, Investigation, Methodology, Resources, Writing – review & editing. XC: Data curation, Formal analysis, Software, Writing – review & editing. XZ: Data curation, Formal analysis, Software, Writing – review & editing. YM: Data curation, Investigation, Methodology, Resources, Writing – review & editing. YZ: Conceptualization, Formal analysis, Funding acquisition, Investigation, Methodology, Project administration, Resources, Software, Supervision, Validation, Writing – review & editing. TZ: Conceptualization, Formal analysis, Funding acquisition, Investigation, Methodology, Project administration, Resources, Supervision, Writing – review & editing. YLZ: Conceptualization, Formal analysis, Investigation, Methodology, Software, Supervision, Validation, Visualization, Writing – review & editing.

## References

[1] Brandt C Zvolensky MJ Woods SP Gonzalez A Safren SA O’Cleirigh CM. Anxiety symptoms and disorders among adults living with HIV and aids: A critical review and integrative synthesis of the empirical literature. Clin Psychol Rev. (2017) 51:164–84. doi: 10.1016/j.cpr.2016.11.005 PMC5195877 27939443

[2] Treisman GJ Soudry O. Neuropsychiatric effects of HIV antiviral medications. Drug Saf. (2016) 39:945–57. doi: 10.1007/s40264-016-0440-y 27534750

[3] Organization AP. Diagnostic and Statistical Manual of Mental Disorders, 5th Ed. Virginia, United States: American Psychiatric Publishing, Inc (2013).

[4] Evaluation IoHMa. In: Global Health Data Exchange (Ghdx). Population Health Building/Hans Rosling Center 3980 15th Ave. NE, Seattle, WA 98195 United States: Institute for Health Metrics and Evaluation Available at: https://vizhub.healthdata.org/gbd-results/.

[5] Adeli E Zahr NM Pfefferbaum A Sullivan EV Pohl KM. Novel machine learning identifies brain patterns distinguishing diagnostic membership of human immunodeficiency virus, alcoholism, and their comorbidity of individuals. Biol Psychiatry Cogn Neurosci Neuroimaging. (2019) 4:589–99. doi: 10.1016/j.bpsc.2019.02.003 PMC6556407 30982583

[6] Pfefferbaum A Zahr NM Sassoon SA Kwon D Pohl KM Sullivan EV. Accelerated and premature aging characterizing regional cortical volume loss in human immunodeficiency virus infection: contributions from alcohol, substance use, and hepatitis C coinfection. Biol Psychiatry Cogn Neurosci Neuroimaging. (2018) 3:844–59. doi: 10.1016/j.bpsc.2018.06.006 PMC6508083 30093343

[7] Tesic T Boban J Bjelan M Todorovic A Kozic D Brkic S. Basal ganglia shrinkage without remarkable hippocampal atrophy in chronic aviremic HIV-positive patients. J Neurovirology. (2018) 24:478–87. doi: 10.1007/s13365-018-0635-3 29687405

[8] O’Connor EE Zeffiro T Lopez OL Becker JT Zeffiro T. HIV infection and age effects on striatal structure are additive. J Neurovirology. (2019) 25:480–95. doi: 10.1007/s13365-019-00747-w PMC10488234 31028692

[9] Ances BM Hammoud DA. Neuroimaging of HIV-associated neurocognitive disorders (Hand). Curr Opin HIV AIDS. (2014) 9:545–51. doi: 10.1097/coh.0000000000000112 PMC4217490 25250553

[10] Wang Q Wang C Deng Q Zhan L Tang Y Li H. Alterations of regional spontaneous brain activities in anxiety disorders: A meta-analysis. J Affect Disord. (2022) 296:233–40. doi: 10.1016/j.jad.2021.09.062 34619449

[11] Xu J Van Dam NT Feng C Luo Y Ai H Gu R. Anxious brain networks: A coordinate-based activation likelihood estimation meta-analysis of resting-state functional connectivity studies in anxiety. Neurosci Biobehav Rev. (2019) 96:21–30. doi: 10.1016/j.neubiorev.2018.11.005 30452934

[12] LeDoux JE Pine DS. Using neuroscience to help understand fear and anxiety: A two-system framework. Am J Psychiatry. (2016) 173:1083–93. doi: 10.1176/appi.ajp.2016.16030353 27609244

[13] Geng H Li X Chen J Li X Gu R. Decreased intra- and inter-salience network functional connectivity is related to trait anxiety in adolescents. Front Behav Neurosci. (2015) 9:350. doi: 10.3389/fnbeh.2015.00350 26834594 PMC4720749

[14] He Y Xu T Zhang W Zuo XN. Lifespan anxiety is reflected in human amygdala cortical connectivity. Hum Brain Mapp. (2016) 37:1178–93. doi: 10.1002/hbm.23094 PMC5064618 26859312

[15] Najjar S Pearlman DM Alper K Najjar A Devinsky O. Neuroinflammation and psychiatric illness. J Neuroinflamm. (2013) 10:43. doi: 10.1186/1742-2094-10-43 PMC3626880 23547920

[16] Loomba-Albrecht LA Bregman T Chantry CJ. Endocrinopathies in children infected with human immunodeficiency virus. Endocrinol Metab Clinics North America. (2014) 43:807–28. doi: 10.1016/j.ecl.2014.06.001 25169569

[17] Leonardo ED Hen R. Genetics of affective and anxiety disorders. Annu Rev Psychol. (2006) 57:117–37. doi: 10.1146/annurev.psych.57.102904.190118 16318591

[18] Wallace DR. HIV-associated neurotoxicity and cognitive decline: therapeutic implications. Pharmacol Ther. (2022) 234:108047. doi: 10.1016/j.pharmthera.2021.108047 34848202

[19] Miller AH Raison CL. The role of inflammation in depression: from evolutionary imperative to modern treatment target. Nat Rev Immunol. (2016) 16:22–34. doi: 10.1038/nri.2015.5 26711676 PMC5542678

[20] Danese A Caspi A Williams B Ambler A Sugden K Mika J. Biological embedding of stress through inflammation processes in childhood. Mol Psychiatry. (2011) 16:244–6. doi: 10.1038/mp.2010.5 PMC4212809 20157309

[21] Leserman J. HIV disease progression: depression, stress, and possible mechanisms. Biol Psychiatry. (2003) 54:295–306. doi: 10.1016/s0006-3223(03)00323-8 12893105

[22] Button KS. Double-dipping revisited. Nat Neurosci. (2019) 22:688–90. doi: 10.1038/s41593-019-0398-z 31011228

[23] Holzschneider K Mulert C. Neuroimaging in anxiety disorders. Dialogues Clin Neurosci. (2011) 13:453–61. doi: 10.31887/DCNS.2011.13.4/kholzschneider PMC3263392 22275850

[24] Sewards TV Sewards MA. On the neural correlates of object recognition awareness: relationship to computational activities and activities mediating perceptual awareness. Consciousness Cogn. (2002) 11:51–77. doi: 10.1006/ccog.2001.0518 11883988

[25] Ishai A Ungerleider LG Martin A Schouten JL Haxby JV. Distributed representation of objects in the human ventral visual pathway. Proc Natl Acad Sci USA. (1999) 96:9379–84. doi: 10.1073/pnas.96.16.9379 PMC17791 10430951

[26] Baddeley R Abbott LF Booth MC Sengpiel F Freeman T Wakeman EA. Responses of neurons in primary and inferior temporal visual cortices to natural scenes. Proc Biol Sci. (1997) 264:1775–83. doi: 10.1098/rspb.1997.0246 PMC1688734 9447735

[27] Iidaka T Omori M Murata T Kosaka H Yonekura Y Okada T. Neural interaction of the amygdala with the prefrontal and temporal cortices in the processing of facial expressions as revealed by fMRI. J Cogn Neurosci. (2001) 13:1035–47. doi: 10.1162/089892901753294338 11784442

[28] Goldin PR Manber-Ball T Werner K Heimberg R Gross JJ. Neural mechanisms of cognitive reappraisal of negative self-beliefs in social anxiety disorder. Biol Psychiatry. (2009) 66:1091–9. doi: 10.1016/j.biopsych.2009.07.014 PMC2788040 19717138

[29] Li C Su S Wu H Zhu Y. Abnormal spontaneous brain activity in patients with generalized anxiety disorder revealed by resting-state functional MRI. Neuroreport. (2018) 29:397–401. doi: 10.1097/wnr.0000000000000982 29406370

[30] Xia L Li S Wang T Guo Y Meng L Feng Y. Spontaneous alterations of regional brain activity in patients with adult generalized anxiety disorder. Neuropsychiatr Dis Treat. (2017) 13:1957–65. doi: 10.2147/ndt.S133853 PMC5530096 28790831

[31] Serra-Blasco M Radua J Soriano-Mas C Gómez-Benlloch A Porta-Casteràs D Carulla-Roig M. Structural brain correlates in major depression, anxiety disorders and post-traumatic stress disorder: A voxel-based morphometry meta-analysis. Neurosci Biobehav Rev. (2021) 129:269–81. doi: 10.1016/j.neubiorev.2021.07.002 34256069

[32] Nichols MJ Gates TM Soares JR Moffat KJ Rae CD Brew BJ. Atrophic brain signatures of mild forms of neurocognitive impairment in virally suppressed HIV infection. AIDS (London England). (2019) 33:55–66. doi: 10.1097/qad.0000000000002042 30325766

[33] Archibald SL Masliah E Fennema-Notestine C Marcotte TD Ellis RJ McCutchan JA. Correlation of *in vivo* neuroimaging abnormalities with postmortem human immunodeficiency virus encephalitis and dendritic loss. Arch Neurol. (2004) 61:369–76. doi: 10.1001/archneur.61.3.369 15023814

[34] Uddin LQ Yeo BTT Spreng RN. Towards a universal taxonomy of macro-scale functional human brain networks. Brain Topography. (2019) 32:926–42. doi: 10.1007/s10548-019-00744-6 PMC7325607 31707621

[35] Carey G Görmezoğlu M de Jong JJA Hofman PAM Backes WH Dujardin K. Neuroimaging of anxiety in parkinson’s disease: A systematic review. Movement disorders: Off J Movement Disord Soc. (2021) 36:327–39. doi: 10.1002/mds.28404 PMC7984351 33289195

[36] Kreifelts B Weigel L Ethofer T Brück C Erb M Wildgruber D. Cerebral resting state markers of biased perception in social anxiety. Brain Structure Funct. (2019) 224:759–77. doi: 10.1007/s00429-018-1803-1 30506458

[37] Zhu H Qiu C Meng Y Yuan M Zhang Y Ren Z. Altered topological properties of brain networks in social anxiety disorder: A resting-state functional MRI study. Sci Rep. (2017) 7:43089. doi: 10.1038/srep43089 28266518 PMC5339829

[38] Ding J Chen H Qiu C Liao W Warwick JM Duan X. Disrupted functional connectivity in social anxiety disorder: A resting-state fMRI study. Magnetic resonance Imaging. (2011) 29:701–11. doi: 10.1016/j.mri.2011.02.013 21531100

[39] Etkin A Prater KE Schatzberg AF Menon V Greicius MD. Disrupted amygdalar subregion functional connectivity and evidence of a compensatory network in generalized anxiety disorder. Arch Gen Psychiatry. (2009) 66:1361–72. doi: 10.1001/archgenpsychiatry.2009.104 PMC12553334 19996041

[40] Zugman A Jett L Antonacci C Winkler AM Pine DS. A systematic review and meta-analysis of resting-state fMRI in anxiety disorders: need for data sharing to move the field forward. J Anxiety Disord. (2023) 99:102773. doi: 10.1016/j.janxdis.2023.102773 37741177 PMC10753861

[41] Frauenschuh A DeVico AL Lim SP Gallo RC Garzino-Demo A. Differential polarization of immune responses by co-administration of antigens with chemokines. Vaccine. (2004) 23:546–54. doi: 10.1016/j.vaccine.2004.06.024 15530704

[42] Michopoulos V Powers A Gillespie CF Ressler KJ Jovanovic T. Inflammation in fear- and anxiety-based disorders: PTSD, GAD, and beyond. Neuropsychopharmacology: Off Publ Am Coll Neuropsychopharmacol. (2017) 42:254–70. doi: 10.1038/npp.2016.146 PMC5143487 27510423

[43] Nixon DE Landay AL. Biomarkers of immune dysfunction in HIV. Curr Opin HIV AIDS. (2010) 5:498–503. doi: 10.1097/COH.0b013e32833ed6f4 20978393 PMC3032605

[44] Justice AC Freiberg MS Tracy R Kuller L Tate JP Goetz MB. Does an index composed of clinical data reflect effects of inflammation, coagulation, and monocyte activation on mortality among those aging with HIV? Clin Infect diseases: an Off Publ Infect Dis Soc America. (2012) 54:984–94. doi: 10.1093/cid/cir989 PMC3297653 22337823

[45] Olsen D Kaas M Schwartz O Nykjaer A Glerup S. Loss of bdnf or its receptors in three mouse models has unpredictable consequences for anxiety and fear acquisition. Learn Memory. (2013) 20:499–504. doi: 10.1101/lm.032045.113 23959707

[46] Potts NL Davidson JR Krishnan KR Doraiswamy PM. Magnetic resonance imaging in social phobia. Psychiatry Res. (1994) 52:35–42. doi: 10.1016/0165-1781(94)90118-X 8047620

[47] Grinspoon SK Bilezikian JP. HIV disease and the endocrine system. New Engl J Med. (1992) 327:1360–5. doi: 10.1056/nejm199211053271906 1406838

