# Supplementary material for: Brain function abnormalities and neuroinflammation in people living with HIV-associated anxiety disorders
Source: Front Psychiatry. 2024 Mar 18;15:1336233. doi: 10.3389/fpsyt.2024.1336233 (PMC10984160; doi:10.3389/fpsyt.2024.1336233)
Supplement: Supplementary file 1 [file Table_1.docx]

Supplementary Material

# Supplementary Tables

Supplementary Table 1 Functional alterations in HIV-associated anxiety disorders

| Brain functional and structural indexes | Brain ares | AAL area | MNI coordinates (X, Y, Z) mm | Number of voxels | Peak t-value | Cluster-level P value | Defination of ROI |
| --- | --- | --- | --- | --- | --- | --- | --- |
| ALFF | Temporal_Inf_R | 90 | 68, -18, -27 | 13 | 6.5087 | 0.017 (uncorrected) | ROI 1 |
| ReHo | Occipital_Sup_R | 50 | 15, -87, 36 | 11 | 3.9617 | 0.104 (uncorrected) | ROI 2 |

The differences in ALFF and ReHo between HIV control and HIV ANXs were performed with a two-sample t-test. AAL areas of the cerebral cortex are defined by cytoarchitecture, proposed by the German anatomist Korbinian Brodmann in the early 1900’s MNI coordinates represents the spatial coordinate position of the brain area. It refer to the normalized space defined in SPM12, which is in mm, and the (X, Y, Z) axes are oriented towards (right, anterior, posterior). The origin is at the AC (anterior commissure) point of the template, with the (negative) Y axis also going through the PC (posterior commissure) point of the template Number of voxels represent the size of brain areas. ROI: region of interest; ALFF: amplitude of low-frequency fluctuations; ReHo: Regional Homogeneity; Temporal_Inf_R: the right inferior temporal gyrus; Occipital_Sup_R: the right superior occipital gyrus

Supplementary Table2 Seed-based functional connectivity differences between HIV control and HIV ANXs

| ROI | Seed | Connected areas | MNI coordinates (X, Y, Z) mm | Number of voxels | Peak t-value | Alterations | Cluster-level P value |
| --- | --- | --- | --- | --- | --- | --- | --- |
| 1 | Temporal_Inf_R | Occipital_Mid_R | 33， -90， 0 | 12 | 4.0597 | Decrease | 0.129 |
|  |  | undefined | -15, 48, 12 | 14 | 4.5985 | Decrease | 0.103 |
|  |  | Occipital_Sup_R | 27, -84, 33 | 39 | 4.3205 | Decrease | 0.011 |
|  |  | Postcentral_L | -54, 21, 54 | 13 | 4.7309 | Decrease | 0.115 |
|  |  | Thalamus_L | -3, -18, 12 | 12 | 4.2505 | Increase | 0.083 |
| 2 | Occipital_Sup_R | Temporal_Inf_L | -57, -54, -9 | 11 | 3.8145 | Increase | 0.149 |
| 13 | Left hippocampus | Frontal_Sup_Medial_R | 6, 39, 51 | 11 | 5.4887 | Increase | 0.011 |
|  |  | Supp_Motor_Area_L | 0, 21, 63 | 10 | 4.1321 | Increase | 0.019 |

Set brain regions with significant ALFF and ReHo differences as seeds for FC analysis. Altered ALFF in the right inferior temporal gyrus (ROI 1), right superior occipital gyrus (ROI 2) The differences in FC between HIV ANXs and HIV control were performed with a two-sample t-test. The meaning of MNI coordinates and Number of voxels are the same as Table 2. Temporal_Inf_R: the right inferior temporal gyrus; Occipital_Sup_R: the right superior occipital gyrus. Occipital_Mid_R: the right middle occipital gyrus; Occipital_Sup_R: the right superior occipital gyrus; Postcentral_L: the left postcentral gyrus; Thalamus_L: the left thalamus; Temporal_Inf_L: the left inferior temporal gyrus; Frontal_Sup_Medial_R: the right superior frontal gyrus, medial orbita.l Supp_Motor_Area_L: the left supplementary motor area.

Supplementary Table3 ROI definition for function connectivity analysis

| ROI | Seed |
| --- | --- |
| 1 | Temporal_Inf_R |
| 2 | Occipital_Sup_R |
| 3 | Frontal_Sup_Orb_L |
| 4 | Frontal_Sup_Orb_R |
| 5 | Frontal_Mid_L |
| 6 | Frontal_Mid_R |
| 7 | Frontal_Mid_Orb_L |
| 8 | Frontal_Mid_Orb_R |
| 9 | Frontal_Inf_Orb_L |
| 10 | Frontal_Inf_Orb_R |
| 11 | Frontal_Mid_Orb_L |
| 12 | Frontal_Mid_Orb_R |
| 13 | Hippocampus_L |
| 14 | Hippocampus_R |
| 15 | Caudate_L |
| 16 | Caudate_R |
| 17 | Putamen_L |
| 18 | Putamen_R |
| 19 | Pallidum_L |
| 20 | Pallidum_R |
| 21 | Thalamus_L |
| 22 | Thalamus_R |

ROI: region of interest; R: right; L: left; Temporal_Inf: inferior temporal gyrus; Occipital_Sup: superior occipital gyrus; Frontal_Sup_Orb: superior frontal gyrus, orbital part; Frontal_Mid: middle frontal gyrus; Frontal_Mid_Orb: middle frontal gyrus, orbital part; Frontal_Inf_Orb: inferior frontal gyrus, orbital part;

Supplementary Table 4 Multivariate analysis of variance (MANOVA)

| Independent Variables | Mean Squares | F | *P* value |
| --- | --- | --- | --- |
| Eotaxin | 181.244 | 1.595 | 0.211 |
| G_CSF | 1047.138 | 0.865 | 0.356 |
| IL_8 | 0.485 | 0.396 | 0.531 |
| IL_12 | 716.315 | 1.331 | 0.253 |
| IL_18 | 46247.724 | 1.617 | 0.208 |
| IP_10 | 177.174 | 0.019 | 0.891 |
| MCP_1 | 4789.658 | 1.822 | 0.182 |
| MIG | 1739904.139 | 2.946 | 0.091 |
| MIP_1_beta | 80.195 | 0.109 | 0.743 |
| VEGF_A | 10030.780 | 1.206 | 0.276 |
| ACTH | 1.037 | 0.801 | 0.374 |
| CORT | 29.811 | 0.385 | 0.537 |
| CRHBP | 0.100 | 0.079 | 0.779 |
| GDNF | 103.150 | 0.303 | 0.584 |

G-CSF: granulocyte colony-stimulating factor; IL: interleukin; MCP: monocyte chemotactic protein; MIP: macrophage inflammatory protein; VEGF: vascular endothelial growth factor; ACTH: adrenocorticotropic-hormone; CORT: cortisol; Dependent variables: anxiety disorders diagnosis by DSM-5. *: p<0.05.
